# Effects of a remote-handling-concept–based task-oriented arm training (ReHab-TOAT) on arm-hand skill performance in chronic stroke: a study protocol for a two-armed randomized controlled trial

**DOI:** 10.1186/s13063-023-07139-w

**Published:** 2023-03-14

**Authors:** Jule Elmanowski, Henk Seelen, Richard Geers, Melanie Kleynen, Jeanine Verbunt

**Affiliations:** 1 Department of Rehabilitation Medicine, Care and Public Health Research Institute, Maastricht University, Maastricht, the Netherlands; 2 Adelante Centre of Expertise in Rehabilitation and Audiology, Hoensbroek, the Netherlands; 3 Adelante Rehabilitation Centre, Hoensbroek, the Netherlands; 4 Research Centre for Nutrition, Lifestyle and Exercise, Faculty of Health, Zuyd University of Applied Sciences, Heerlen, the Netherlands

**Keywords:** Stroke, Rehabilitation, Functional performance, Upper limb, Technology, Haptic feedback, Randomized clinical trial, Effectiveness, Clinical study, Clinical trial protocol

## Abstract

**Background:**

Improving arm-hand skill performance is a major therapeutic target in stroke rehabilitation and needs intensive and varied training. However, guided treatment time is limited. Technology can assist in the training of patients, offering a higher intensity and more variety in content. A new task-oriented arm training approach was developed, using a ‘Remote Handling concept based’ device to provide haptic feedback during the performance of daily living activities (ReHab-TOAT). This study aims to investigate the effects of ReHab-TOAT on patients’ arm-hand function and arm-hand skill performance, quality of life of both patients in the chronic phase after stroke and their caregivers and the patients’ perception regarding the usability of the intervention.

**Methods:**

A randomized clinical trial was designed. Adult chronic stroke patients suffering from hemiparesis and arm-hand problems, with an Utrechtse Arm-hand Test score of 1–3, will be invited to participate. Participants in the experimental group receive ReHab-TOAT additional to care as usual. ReHab-TOAT contains task-oriented arm training for stroke patients in combination with haptic feedback, generated by a remote handling device. They will train for 4 weeks, 3× per week, 1.5h per day. Participants in the control group will receive no additional therapy apart from care as usual. The Fugl-Meyer Assessment (FMA), measuring participants’ motor performance of the affected arm, is used as the primary outcome measure. Secondary outcome measures are arm-hand capacity of the patient (ARAT), perceived arm-hand skill performance (MAL), actual arm-hand skill performance (accelerometry), patients’ quality of life (EuoQol-5D) and caregivers’ quality of life (CarerQoL). Participants’ perception regarding the usability of the intervention, including both the developed approach and technology used, will be evaluated by the System Usability Scale and a questionnaire on the user experience of technology. Measurements will be performed at 1, 2, 3 and 4 weeks pre-intervention (baseline); immediately post-intervention; and 3, 6 and 9 months post-intervention. Statistical analysis includes linear mixed model analysis.

**Discussion:**

This study is designed to investigate the evidence regarding the effects of ReHab-TOAT on patients’ performance at different levels of the *I*nternational *C*lassification of *F*unctioning, disability and health (*ICF*) model, i.e. a framework measuring functioning and disability in relation to a health condition, and to provide insights on a successful development and research process regarding technology-assisted training in co-creation.

**Trial registration:**

Netherlands Trial Register NL9541. Registered on June 22, 2021

**Supplementary Information:**

The online version contains supplementary material available at 10.1186/s13063-023-07139-w.

## Administrative information

Note: the numbers in curly brackets in this protocol refer to SPIRIT checklist item numbers. The order of the items has been modified to group similar items (see http://www.equator-network.org/reporting-guidelines/spirit-2013-statement-defining-standard-protocol-items-for-clinical-trials/).

**Table:** 

Title {1)	Effects of a remote-handling-concept based task-oriented arm training (ReHab-TOAT) on arm-hand skill performance in chronic stroke: a study protocol for a two-armed randomized controlled trial
Trial registration {2a and 2b}	Netherlands Trial Register: NL9541
Protocol version {3}	19-12-2022 Version 2
Funding {4}	This study is investigator initiated and is funded by Adelante Zorggroep as a sponsor.
Author details {5a}	^1^ Maastricht University, Care and Public Health Research Institute, Department of Rehabilitation Medicine, Maastricht, the Netherlands ^2^ Adelante Centre of Expertise in Rehabilitation and Audiology, Hoensbroek, the Netherlands ^3^ Adelante Rehabilitation Centre, Hoensbroek, the Netherlands ^4^ Zuyd University of Applied Sciences, Research Centre for Nutrition, Lifestyle and Exercise, Faculty of Health, Heerlen, the Netherlands §Corresponding author
Name and contact information for the trial sponsor {5b}	Adelante Zorggroep Henri Plagge Zandbergsweg 111 6432CC Hoensbroek Netherlands
Role of sponsor {5c}	Adelante Zorggroep has a sponsor role in the form of salaries of the authors JE, RG, JV and HS. Zuyd University of Applied Sciences supports this study in the form of salary of the author MK. The funding bodies do not have a role in the design of the study and collection, analysis and interpretation of data, and in writing of the manuscript.

## Introduction

### Background and rationale

Sixty-six percent of stroke patients still experience deficits in the upper limb 6 months after stroke [1], and only 5 to 20% reach complete functional recovery of the paretic arm in terms of arm-hand skill performance (AHSP) [1, 2]. It is hypothesized that more motor therapy time and higher variability in treatment content may lead to further improvement [3, 4], which next to improved quality of life of the patient may also lead to improved quality of life of his/her caregiver, because both the amount and content of support for the patient may decrease/change.

Current rehabilitation approaches are based on the International Classification of Functioning, disability and health (ICF) [5], describing human functioning at three different levels, i.e. ‘function’ level related to body functions and structures, ‘activity’ level related to task/skill execution and ‘participation’ level related to patients’ involvement in social life situations [6–9]. There is a growing body of evidence that training at the level of activities (e.g. task-oriented) results in improvements at both the level of body functions and the level of activity, whereas training solely at the level of body functions (e.g. isolated strength training) does not necessarily result in improvements at the level of activities [10, 11]. Furthermore, in contrast to many beliefs and dogmas in rehabilitation, persons in the chronic stage after a stroke may still have the ability to improve on arm-hand function (AHF), AHSP and actual use of the affected arm in daily life. Research has shown a 43% improvement in the amount of use of the affected arm in chronic stroke patients after an 8-week sensor-based treatment (T-TOAT) [12, 13].

#### Task-oriented arm training (TOAT)

One of the training approaches focusing on improvements on the ICF domains of activities and participation is task-oriented training [8, 14] in which the patient trains meaningful activities in a functional context [13, 15, 16]. According to theories on motor control and training physiology [17, 18], these meaningful activities may be broken down into so-called sub-activities or dominant phases (=task segmentation) and then be trained separately (=part-practise), after which the sub-activities will be ‘re-assembled’ (=chaining) towards the complete skill again [17–20].

Furthermore, feedback is essential to induce learning of motor skills. The internal feedback mechanism is often affected in stroke patients [21, 22]. Therefore, external feedback is important to gain improvements during training. One example of external feedback is haptic feedback [23, 24]. Haptic feedback is often provided by therapists using their own hands, since haptic feedback may lead to improvements on the coordination of movements and thereby also on AHSP. However, for therapists, it is nearly impossible to tailor haptic feedback towards the individual needs of the patient in a reproducible way. Research has shown that task-oriented training using the principles of motor control with sufficient practice time (repetition) may induce neuroplasticity, resulting in skill acquisition as well as retention and transfer of the newly learned skills towards similar skills (generalization), thus making learned strategies available for future behaviour [25–30].

Another advantage of task-oriented training is that the intrinsic motivation of the patient is stimulated by the use of meaningful individual person-oriented training goals related to daily living activities [31], which, in turn, is of great benefit for motor learning [32, 33] and exercise compliance [34, 35].

#### Technology-supported task-oriented arm training (T-TOAT)

In view of the high percentage of patients suffering arm-hand problems after stroke, and the limited treatment time available, new technology is being developed to assist the training of patients. Furthermore, it is well established that a high intensity of practice results in more stable improvements also after training [23]. By using technology-assisted training, AHF training and AHSP training may be augmented both in amount and duration of training as well as in content variety and task specificity. However, for a technology-assisted training to elicit significant and clinically relevant improvements of AHSP in chronic stroke patients, a combination with task-oriented training, like in the T-TOAT approach, is necessary [36]. However, evidence-based, technology-assisted training programmes for stroke patients with an Utrechtse Arm-hand Test (UAT) score [37] of 1 or 2 in the chronic phase after stroke are lacking, even though research has shown that significant improvements at function and activity level following technology-assisted treatment are possible in the chronic stage after stroke [38, 39]. The T-TOAT concept is based on principles of training physiology and motor learning, encompassing task segmentation, part-practise, chaining, overlearning, feedback, variability and training load. These ingredients make the T-TOAT concept especially suitable for training complex skills/activities by combining function level and activity level training requirements and using technology [9, 20]. Technology-assisted training may provide a challenging treatment environment, also for patients with a severely affected arm-hand, while keeping the workload for (para-)medical staff and treatment costs manageable [4, 40, 41]. However, results of previous research in the field of technology-assisted upper limb training in stroke report no or only minor improvements on either motor recovery or activities of daily living [42, 43]. A possible explanation for these findings may lay in the fact that the developed technologies are not focussing on training at the level of activities and participation [36].

#### New training approach ‘ReHab-TOAT’

A new task-oriented arm training approach using a so-called remote handling concept, to generate haptic feedback on the patient’s arm, aiming at improvements on arm function level and, ultimately, on the level of activities and participation, was developed. This approach is called ReHab-TOAT—‘Remote Handling concept based, Task-Oriented Arm Training’. ReHab-TOAT may be applicable for a wide variety of patients with arm-hand problems due to a central nervous system deficit. However, as our Rehab-TOAT approach has yet to be evaluated on its effectiveness, we chose to focus specifically on patients in the chronic stage after a stroke, since these patients, in general, receive little therapy related to impaired arm function. Also, these patients are in a more stable phase after stroke, in which spontaneous recovery is minimal. Furthermore, since ReHab-TOAT’s effectiveness has not yet been established, providing such therapy to patients in the subacute stage, thus replacing other forms of therapy, in this stage of development, is ethically unacceptable.

ReHab-TOAT uses the principles of T-TOAT in combination with assistive forces to generate enriched haptic feedback. Furthermore, ReHab-TOAT uses strategies to improve self-efficacy and self-confidence to generalize and transfer training results to different contexts.

We hypothesize that:

- The use of haptic feedback during task-oriented training may lead to improvements of both arm function and arm skill performance in patients in the chronic stage after a stroke suffering from a moderately to severely affected arm.
- The quality of life of the patient will improve.
- The quality of life of the caregivers will improve as a result of a reduced need to support the patient in daily life tasks.
- These improvements will last beyond the training phase, because patients will continue to use their affected arm in daily life more, thus creating optimal conditions for generalization and transfer of ReHab-TOAT training effects towards other daily tasks performed in different contexts.
- Perceived usability of ReHab-TOAT is high, since the training approach and software are developed in co-creation with therapists, physicians and patients in order to meet their needs and expectations.

### Objectives

The aim of this RCT is to investigate the effects of a 4-week ReHab-TOAT approach in addition to care as usual on improving arm function and arm-hand skill performance in chronic stroke patients with a moderately to severely affected arm-hand up to 9 months after training, compared to only care as usual. Furthermore, the aim of this study is to assess improvements in patients’ and their caregivers’ perceived quality of life, as well as the patients’ perception regarding the usability of the intervention, including both the developed approach and technology used.

## Methods: participants, interventions and outcomes

This RCT uses the SPIRIT reporting guidelines [44]. An overview of the SPIRIT checklist can be found in an Additional file.

### Study setting

This RCT will be performed at Adelante rehabilitation centre, a specialized rehabilitation clinic in the South of the Netherlands. The intervention of this RCT will be performed by a trained physical therapist or occupational therapist with experience in the treatment of patients with neurological disorders, together with a trained senior technician, all having been trained in providing the ReHab-TOAT approach.

### Recruitment and informed consent

Participants will be recruited from the electronic patient database of Adelante rehabilitation centre in Hoensbroek, the Netherlands, or via information flyers published on the website of Adelante rehabilitation centre and via a patient organization for stroke patients. Potential participants will be identified by the rehabilitation specialists of the stroke units, who will perform a preliminary eligibility screening. Potential participants reached via the website of the rehabilitation centre or the patient organization will be preliminary screened for eligibility during a phone call by a member of the research team. This will be done after the person has sent an email with his/her contact details and a declaration of interest in participation in this study. All potential eligible participants will receive an information letter with details on this study. They will be invited for a full screening by a member of the research team. During this appointment, the arm-hand function is assessed as are all inclusion criteria, and an informed consent form is signed by the patient. Similarly, the potential participant’s identified caregiver will receive a study information letter and will be asked to sign an informed consent form too, prior to his/her inclusion into the study.

### Eligibility criteria

Adult stroke patients who are currently in the chronic phase after stroke (i.e. post-stroke time longer than 12 months), who suffer from a hemiplegic arm motor impairment with an UAT score between 1 and 3 [37] and who are able to understand and execute a 3-stage verbal command are included in this study. Participants are excluded if they suffer severe non-stroke-related co-morbidity that may interfere with arm-hand function or interfering spasticity in the affected upper limb, i.e. a Modified Ashworth Scale (MAS) score >= 1+ [45].

Patients’ caregivers are included if they are over 18 years of age and provide informal care to the above-mentioned participants. Patient participants will not be excluded if their caregiver does not want to participate in this study. He/she can ask a different caregiver or decide to participate without any caregiver taking part in the study.

### Trial design and timeline

This single-blinded RCT features two arms (EXP and CONTR). The EXP group will receive a 4-week ReHab-TOAT regime additional to any care the participants may receive outside the research context. The EXP group will also receive 1 additional session to familiarize themselves with the training system, prior to the start of the training phase. The CONTR group will not receive additional arm-hand therapy apart from care as usual at this stage, i.e. therapy participants already may receive from therapists in their current home situation. In our protocol, no restrictions will be imposed on any (additional) therapies participants currently receive.

**Fig. 1:**
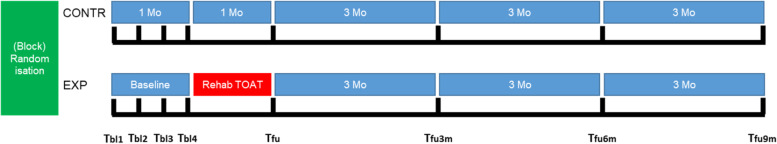
Timeline RCT T.. time of measurement, bl baseline, fu follow-up, EXP experimental group, CONTR control group, m/mo month

In order to assess baseline stability, baseline measurements will be taken weekly during a period of 4 weeks. For both groups, follow-up measurements will be taken after the 4-week period in which the experimental group received ReHab-TOAT, as well as 3, 6 and 9 months thereafter. Figure 1 gives an overview on intervention and measurement time points.

### Randomization

This study will involve 30 chronic stroke patients that will be randomly allocated to either the EXP or the CONTR condition. From a logistical point of view, a maximum of eight participants can join the project at one time, i.e. in order of inclusion. This results in four sets of participants, three sets of 8 participants and one set of 6 participants. Allocation to the EXP or CONTR condition will then be randomly assigned for all participants within one set at the same time. Randomization will be performed as follows: Based on the set size, equal numbers of closed, opaque envelopes, each containing a white piece of paper containing either the acronym ‘EXP’ or ‘CONTR’, are scrambled by a researcher blinded for patient information. Subsequently, another researcher blinded for patient information will randomly draw separate envelopes from this stack, each of which will then, without having been opened, be allocated to an anonym participant code. After all envelopes within one set have been issued, the content of the opaque envelope and the participant code it was randomly allocated to will be officially recorded by the research coordinator in the presence of the latter blinded researcher. This procedure is repeated for each set of participants subsequently.

### Blinding

Blinding patients and/or therapists for treatment allocation is not possible. One trained researcher who is blinded for therapy modality will perform all measurements.

### Intervention

Participants in the EXP group receive a 4-week training period with the ReHab-TOAT approach at a frequency of 3 sessions of 1.5 h per week. During a previous feasibility and pilot study, a 4-week training period with ReHab-TOAT seemed to be a feasible intervention period in which clinically relevant results could be achieved. An overview of a training session of ReHab-TOAT can be found in Table 1. One session of ReHab-TOAT can be divided into five phases, where the results of the assessment define the subsequent phases, especially the content, and start settings used for the part of training with the remote handling device. Based on patient’s individual needs, the therapist may adapt this training content and parameters like feedback, intensity and extent continuously during the session [46–48].

**Table 1:** ReHab-TOAT session

Phase	Elements	Aim of the phase	Approximate timing within the sessions
Assessment	• Patient performs 4 activities of daily living under real-life conditions • Skill analysis by PT or OT • Patient rates performance using VAS	• Skill analysis • Choice of activities that will be practised for 1 week (done at the start of each week)	10 min
Preparation	• Preparative exercises for arm and shoulder joints and muscles	• Making the arm and shoulder flexible/supple	10 min
Training with haptic feedback	• Patient performs sets of exercises • Task segmentation is used (first isolated components are trained, later combinations (chaining)) • Dexter^TM^ is used to generate haptic feedback	• Improving (the components of) the activities chosen	50 min with resting periods in-between
Re-assessment	• Patient performs the 4 activities of daily living under real-life conditions • Patient rates performance using VAS • Tips on performing the activities in the home environment	• Skill analysis • Motivation patient • Encourage practice in real-life circumstances	10 min
Homework	• Strength and mobility exercises • Continuing using their affected arm in discussed daily activities	• Encourage practice in real-life circumstances	Based on individual needs

*PT* physical therapist, *OT* occupational therapist, *VAS* visual analogue scale

The main goal of ReHab-TOAT is for the patient to obtain an independent use of the affected arm during the training sessions but especially in other activities and situations, like the home situation of the patient. Therefore, one aspect in all phases of ReHab-TOAT is to facilitate the generalization of training effects towards other/different activities and contexts. Generalization is obtained through various strategies, for example by creating different contexts during practice, giving advice during training and also for non-trained activities and situations, emphasizing achievements during training to raise self-confidence in using the affected arm-hand, discussing and evaluating homework assignments with the patient and caregivers to stimulate training and using the affected arm without supervision and in different contexts [49–54].

**Fig. 2:**
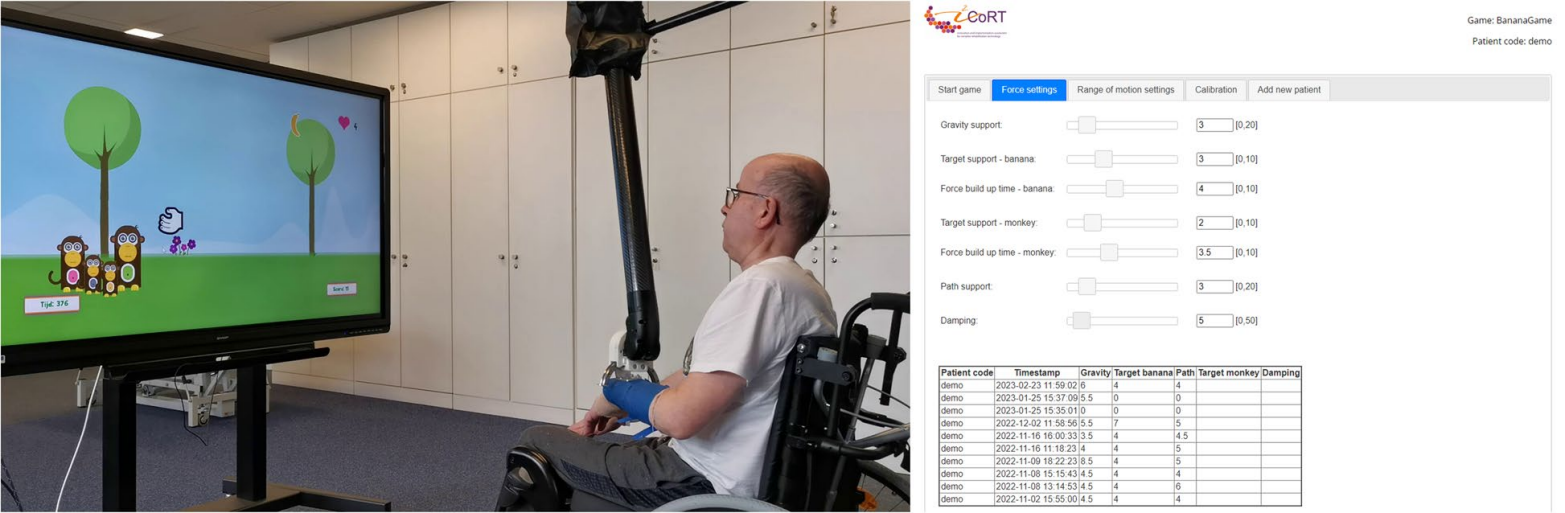
Relationship between Dexter^TM^ and exercises/games on screen and therapist interface

During the training phase with haptic feedback, a remote handling device with bespoke software is used (Fig. 2). This device is called Dexter™ (developed by Veolia Nuclear Solutions UK, Didcot, UK). It is an advanced remote manipulator system which is used in a master-slave construction to replicate the flexible, fine motor function of humans in an environment where humans cannot go, like nuclear waste disposal sites. It can be used to manipulate proprioception by giving haptic feedback to the arm of the participant in six degrees of freedom, resulting in a high range of motion for the user (see the Appendix for more information). A software-driven interface was designed by the Expertise centre for Digital Media (EDM) of Hasselt University (Belgium) in collaboration with Adelante, to facilitate therapists to work with their patients without the need of a technician to be present. However, in this project, for practical and safety reasons, a technician will always be present during the ReHab-TOAT sessions.

The whole intervention is patient-centred and patient-tailored. Furthermore, the therapy is constantly monitored by the therapist and adaptations to the training can be made instantaneous. If changes in one or more of the following factors occur at any moment during the training session, possible adaptations to the training will be discussed with the patient: changes in pain level, changes in movement speed, changes in compensation strategies, changes in strength, changes in movement flexibility and selectivity, changes in range of motion of the arm, changes in muscle fatigue or general fatigue, changes in concentration possibilities, changes in motivation of the patient, changes in perceived fun, changes in the (motor) performance of the tasks and changes in the performance of the exercise (e.g. less repetitions). If both therapist and patient together decide which adaptations are necessary, these adaptations can be made instantaneous on an ‘as needed’ basis, both in terms of training intensity and (skill) training content.

Each training session will be recorded by the therapist in a training log. Information about the training, for example about the sort of preparation exercises used, the intensity of the exercises, session duration and resting periods, feedback given by the robotic device, evaluation of the present training session, evaluation of the time between the training sessions, perceived differences in their home situation, issues in participant-therapist interaction, etc., will be reported. This information will be evaluated at the end of the study. Both therapists involved in the delivery of the training regularly meet to discuss the treatment and progress of patients.

In case of any missed intervention or measurement sessions, the research coordinator will re-schedule this session as soon as possible. Finally, in case a participant wants to stop the intervention, (s)he can stop at any moment without providing the reason behind his/her decision.

### Outcome measures

An overview of all outcome measures and measurement time points can be found in Table 2. The primary outcome of this RCT will be the *motor performance* of the affected arm-hand, measured using the upper extremity section of the Fugl-Meyer Assessment (FMA). The 33 items assess reflexes, movements and coordination of the shoulder, elbow, wrist and hand. The FMA has been found to be reliable and valid in stroke patients [55–57] at the impairment level [56, 58, 59]. The FMA has been used as a gold standard against which other scales have been validated [59].

**Table 2:** Overview measurements and time points

		Study period										
**Timepoints**		**Enrolment**	**Allocation**	**Post-allocation**								
				***Baseline***				***Intervention***	***Follow-up***			
		*T*_*-1*_	*T*_*0*_	*T*_*BL1*_	*T*_*BL2*_	*T*_*BL3*_	*T*_*BL4*_		*T*_*FU*_	*T*_*FU3m*_	*T*_*FU6m*_	*T*_*FU9m*_
**Enrolment**												
Eligibility screening		X										
Informed consent		X										
Allocation			X									
**Interventions**												
ReHab-TOAT (EXP)								X				
Therapy-as-usual (CONTR)								X				
**Assessments**												
Function level	*UAT*		X									
	*MAS*		X									
	*FMA*			X	X	X	X		X	X	X	X
Activity level	*ARAT*			X	X	X	X		X	X	X	X
	*MAL*			X	X	X	X		X	X	X	X
	*Accelerometry (each for 5 consecutive days)*			X					X	X	X	X
Personal factors	*Patient demographics and characteristics*		X									
	*EuroQol-5D*			X					X	X	X	X
External factors	*Single question on any occurred events that may influence measurements*			X	X	X	X		X	X	X	X
	*CarerQol (re. caregiver)*			X					X	X	X	X
	*Single question on amount and content of support (re. caregiver)*			X					X	X	X	X
***Evaluation***												
System Usability Scale									X			
Questionnaire on user experience of technology used				X					X			

*T..* time of measurement, *BL* baseline, *FU* follow-up, *m* month, *Interv* intervention, *EXP* experimental, *CONTR* control, *UAT* Utrechtse Arm Test, *MAS* modified Ashworth Scale, *FMA* Fugl-Meyer Assessment, *ARAT* Action Research Arm Test, *MAL* Motor Activity Log, *SUS* System Usability Scale

Secondary outcome measures will gauge the ICF level of activity and quality of life of the patient and his/her caregiver and will evaluate the usability of the intervention, including both the approach developed and the technology used. Arm-hand *capacity* will be measured using the Action Research Arm Test (ARAT), including grasp movements and reaching movements. The ARAT has been proven to be a reliable, valid and sensitive instrument for upper limb function measurement, which does not show a ceiling effect [60–64]. *Perceived* arm-hand skill performance will be measured using the Motor Activity Log (MAL). The MAL is a semi-structured interview to assess the frequency, quality of use and quality of use of the affected limb during the performance of daily living activities. The MAL has been shown to be a reliable and valid tool for the measurement of arm-hand activity in stroke patients [65]. To measure *actual* arm-hand skill performance, also in different contexts, 3D accelerometers (AX3, Axivity Ltd, UK) are used. Signals from these sensors will be analysed to gauge the actual amount of arm-hand use during daytime, according to the protocols described by Lemmens et al. [66] and Franck et al. [38]. The EuroQol-5D (EQ-5D) will be used to measure the quality of life of the patient regarding perceived health status [67]. The EuroQol-5D has good psychometric properties [68]. To measure the quality of life of the patients’ caregiver, the Care-related Quality of Life instrument (CarerQoL) will be used. It combines a subjective burden measure of the caregiver’s situation with a valuation of personal well-being on a VAS. The CarerQoL has been proven to be feasible, valid and reliable in measuring the impact of caregiving [69–71]. In addition to the CarerQoL, one single question will be asked to measure the amount and content of support the caregiver provides. For evaluation of the used technology and the developed training approach of ReHab-TOAT, the System Usability Scale (SUS) and a self-developed questionnaire will be used. The SUS has been widely used in the evaluation of many systems and is a reliable and valid measure of perceived usability [72–76]. The questionnaire on the user experience of technology used was developed by researchers, technicians and clinicians from Adelante rehabilitation centre and Hasselt University. The questionnaire is based on the Technology Acceptance Model (TAM) and the Unified Theory of Acceptance and Use of Technology (UTAUT) [77] and is established to evaluate the technology-assisted training concept developed. The questionnaire evaluates the perceived benefits, struggles and necessary improvements related to ReHab-TOAT.

Furthermore, a single question gauging the occurrence of any event over the last 2 weeks that may influence the results of the treatment or the measurements (e.g. the patient having had the flu in the past 2 weeks) will be posed. Patients’ demographics and characteristics will be recorded at entry into the study, as will the presence of any restricting form of spasm (Modified Ashworth Scale (MAS)) and AHF status (UAT) [37]. Also, the amount and content of any therapy the participant may potentially receive as care as usual during his/her participation in the project will be recorded in a questionnaire during each measurement session.

### Data collection and management

Data collection of all participants is done by one independent, trained and blinded physical therapist of Adelante rehabilitation centre to eliminate any potential inter-observer differences. All data and results will be handled confidentially. All data will be coded during measurement. Coding will be done using a combination of numeric and alphanumeric characters, which are not related to the participant. Non-coded data (e.g. participant’s name) will be stored separately by the study supervisor. All data from the RCT will be stored in a GDPR-compliant data management system (CASTOR repository (https://www.castoredc.com/; CASTOR, Amsterdam, The Netherlands)). Data entry in Castor is verified by the coordinating researcher, who will also create a locked data file at the end of the study. The coordinating researcher, the study supervisor and the assessing physical therapist have reading access to the data management system. Only the coordinating researcher has full access to the data and the data management system with all rights.

Different retention strategies are used to ensure full data collection and to minimize any withdrawal of participants. The researchers stay in contact with the participants between the training and/or measurement appointments to ask if everything is okay and to kindly remind them of their appointments. Furthermore, the researchers support them with organizing transportation to the appointments and refund the transportation costs quickly. During each appointment, the researchers provide catering for the participants and plan enough time for each session so that nobody feels stressed.

### Sample size

This sample size calculation [78] is based on data gathered in an earlier, small-sized pilot study, featuring pre- and post-intervention measurements, gauging the *order of magnitude* of any potential effect of the ReHab-TOAT treatment in a similar patient group, performed at the Adelante rehabilitation centre. Given the small sample and the skewed distribution, we used a log transformation on those data to estimate the group sizes needed for the RCT.

Given a log-transform of the skewed FMA data gathered during the aforementioned pilot study, a two-sided test and a:

- Mean (log-transformed) pre-intervention FMA value = 1.47 (sd = 0.12)
- Mean post-intervention FMA value = 1.65 (sd = 0.11)
- Power = 0.90
- Alpha = 0.01
- Loss-to-follow-up = 10%

fifteen patient-participants in each group, i.e. 30 participants in total, are needed.

Regarding the patients’ caregivers, 30 persons (associated with the aforementioned patients that are eligible and willing to participate) will also be asked to participate.

### Statistical analysis

#### Primary study parameter(s)

Data from the primary outcome measure (FMA) will be tested for normal distribution using a Kolmogorov–Smirnov test. If normally distributed, data will be statistically analysed using a linear mixed model analysis (factors: GROUP and TIME). If, on the other hand, data are found to be skewed, a log transformation will be performed, after which a linear mixed model will be used to analyse the log-transformed data. Generally, alpha will be set at 0.05. In case of multiple comparison of the data post hoc, a Bonferroni approach will be used to avoid spurious potential false positive findings.

#### Secondary study parameter(s)

The data from the ARAT, the MAL the EuroQol-5D and the CarerQol will be statistically analysed similarly to the data of the FMA. The data regarding patients’ amount of arm-hand use during daytime, derived from the accelerometer signals (according to the protocols described by Lemmens et al. [66] and Franck et al. [38]), will also be statistically analysed similarly to the procedure used to analyse differences in the data of the FMA. Results from the SUS and the questionnaire on user experience of technology used will be reported descriptively. The single questions gauging for any event that may have influenced the patient’s or caregiver’s answer or measurement will only be used to identify/interpret any unexpected measurement and will be reported descriptively. The frequency and content of any additional therapy the participants received during the intervention period will be also reported.

#### Withdrawal of individual subjects, replacement and follow-up

Subjects can leave the study at any time for any reason if they wish to do so, without any consequences. The investigator can decide to withdraw a subject from the study for urgent medical reasons. In the group size calculation, a 10% drop-out rate has been used to compensate for subjects withdrawing from the study.

### Oversight and monitoring

This study (version: V2, dated July 6, 2021) has received ethical approval from the Medical Ethics Committee of Maxima Medical Centre in Veldhoven, the Netherlands (METC reference number: W21.003; CCMO code: NL76382.015.21). All amendments will be notified to the METC that gave a favourable opinion. All substantial amendments will be notified to the METC and to the competent authority. Non-substantial amendments will not be notified to the accredited METC and the competent authority, but will be recorded and filed by the sponsor.

A researcher external to the research team and without competing interest to this RCT is the monitor of the study. During three monitoring sessions, all items of the RCT will be checked following a strict monitoring plan and reported via a monitoring checklist. Additional comments for improvements will be provided by the monitor.

Serious and adverse events (SAE and AE) and potential effects will be reported by the coordinating researcher and the supervisor following a predefined protocol. The study investigator will report all SAEs to the sponsor without undue delay after obtaining knowledge of the events. The sponsor will report the SAEs through the web portal *ToetsingOnline* to the accredited METC that approved the protocol, within 7 days of first knowledge for SAEs that result in death or are life threatening followed by a period of maximum of 8 days to complete the initial preliminary report. All other SAEs will be reported within a period of maximum 15 days after the sponsor has first knowledge of the serious adverse events. All AEs will be followed until they have abated, or until a stable situation has been reached. Depending on the event, follow-up may require additional tests or medical procedures as indicated, and/or referral to the general physician or a medical specialist.

### Dissemination

Results of this RCT will be disseminated through scientific journal publications and conference presentations. Furthermore, the (general) results will be shared with all participants, as well as patient organizations. If the results of this RCT show that ReHab-TOAT is more effective in improving arm-hand function and arm-hand skill performance in chronic stroke patients, the results will be used to create a new arm-hand rehabilitation treatment protocol using remote-handling concepts for clinical implementation in rehabilitation centres. Furthermore, in case of a positive result, we would like to use the results to support the development of a similar yet less expensive, clinically more affordable medical device for the previously mentioned arm-hand rehabilitation treatment. Coded data will be made available to the scientific community upon request.

## Discussion

In this paper, we described the methodology of a randomized controlled single-blinded study that evaluates the effects of ReHab-TOAT on the performance at different levels of the ICF model of persons in the chronic stage after a stroke with an initially moderately to severely affected arm-hand, compared to care as usual.

In the present RCT, the focus lies on a patient subpopulation that is often excluded from research, i.e. on patients with a moderately to severely affected arm. Until recently, treatment options for these patients were limited [79], and even more so for such patients in the chronic stage after stroke. For the latter group, the aim in usual care is to maintain the current arm-hand function status and limit deterioration [10, 80]. However, more recent evidence has been showing that even years after stroke, patients may still gain improvements on a functional level, for example AHF, AHSP and quality as well as the amount of use of the affected arm in daily life [4, 36, 81–83]. The study of Timmermans et al. for example showed improvements of AHF and AHSP exceeding 10% improvement measured using the FMA, ARAT and MAL [20, 36]. If this RCT can underpin these results, the way we look at and organize treatment of patients in the chronic phase may change significantly with consequences for both amount and content of usual care for these patients.

### Methodological considerations

ReHab-TOAT has been developed in a co-creation process with companies, health care professionals, researchers and technicians to create a tailored training approach with suitable software, offering a high amount of possibilities for individualization of training based on the personal needs of the patient and the therapist. The whole development and research process of ReHab-TOAT has been done and described following well-known research protocols. First, the feasibility of ReHab-TOAT was explored in therapists and patients. Next, the order of magnitude of any potential effects in stroke patients was investigated. Subsequently, this RCT was designed and will be performed to investigate the effectiveness of ReHab-TOAT.

For a number of reasons, this study will start out with patients in the chronic stage after a stroke. First, it is assumed that these persons are stable regarding any spontaneous neurological recovery which only occurs in the first month(s) after stroke [84–86]. Any changes in AHF and/or AHSP status in the EXP group relative to baseline values and/or values from the CONTR group may therefore be attributable to the EXP intervention. One might argue that improvement in AHF and/or AHSP in the EXP group relative to the CONTR group may be caused by the amount of therapy provided to the former group. However, we consciously chose to compare our intervention to therapy as usual, because we want to compare it to real-life circumstances and not to any pre-construed circumstances without any knowledge on the effects of content and amount of therapy. Furthermore, there is no evidence that shows that a higher amount of therapy as usual in the chronic phase after stroke will result in substantial improvements. The authors are, however, aware that increased contact time alone, be it for training and/or measurements, may have a benefit on quality of life alone in chronic stroke patients, even without functional improvement of the arm-hand.

Moreover, we hypothesize that, next to AHF and AHSP, the motivation for training as well as the self-efficacy of the patient may increase to due improvements in daily life, which may lead to even higher improvements at function, activity and participation level as well as an increase in quality of life. Therefore, we chose to use measurements on different levels at the ICF model to evaluate the developed training approach in a real-life context.

Furthermore, we also involved the patient’s caregiver in this RCT, because it is known that improvements of the patient will reduce the time and effort the caregiver needs to support the patient in daily life [87]. In many trials, it is hypothesized that improvements of the patient have a positive influence on the caregiver, but there are only a few results due to the few existing measurements. Therefore, we decided to use a single question on the amount and content of provided care next to a worldwide known questionnaire for the QoL of the caregiver.

### Potential consequences for future research

The results regarding the usability of ReHab-TOAT and the technology used may lead to opportunities to further improve and refine both the technology and the developed training programme, and to increase its implementation and use. If ReHab-TOAT is proven to be effective, we can further investigate the dose–response relationship. Regarding the limited treatment time and rising healthcare costs and the intensive treatment time of ReHab-TOAT, it is important to examine which training amount and intensity are needed to optimize the gain in effects, both in the short and the long term. Then, subsequently, a cost-effectiveness study can further investigate the potential reduction of healthcare costs. Such cost-effectiveness study of ReHab-TOAT is also necessary to redevelop the Dexter device into the next generation of smaller and more affordable devices, reducing the total costs for ReHab-TOAT applications, their use in other clinics, and for other target populations.

Moreover, the knowledge and experience gathered in this study may assist in updating current guidelines, may identify important focal points and may assist in identifying (and removing) potential barriers for a successful development and research of novel healthcare technology in co-creation between companies and health care employees.

#### Trial status

Recruitment started in January 2022 and was completed in October 2022. The final participants are expected to complete their assessments in November 2023.

### Supplementary Information

**Additional file 1.** SPIRIT checklist.

## Data Availability

The datasets used and/or analysed during the current study will be made available from the corresponding author upon reasonable request.

## Appendix

### Information on Dexter^TM^

In the following table, an overview of Dexter^TM^ specifications can be found.

Table 3

**Table 3:** Some features of Dexter™ (courtesy: Veolia Nuclear Solutions)

High dexterity	Haptic feedback sensitivity	10 g
	Payload	10 kg
Highly programmable	Weight compensation	• Weight of Dexter™ arm is ‘electronically’ removed • Weight of a tool can be removed
	Force scaling	Force feedback can be increased or decreased
	Active constraints	Use of kinematic feedback to assist tasks, e.g. using a tool
	Virtual walls	A virtual environment can be created
	Guiding trajectories	‘Attraction path’ to guide a movement

The haptic feedback to the patient’s arm, used to facilitate proprioception, is generated by small forces in the range of 0 to 20 N, with a sensitivity of 0.1 N (equivalent to 10 g). Also, if necessary, the patient’s arm may be supported against gravity by weight support provided by DexterTM to a maximum of 7.5 kg

## Abbreviations

AHF: Arm-hand function
AHSP: Arm-hand skill performance
ARAT: Action Research Arm Test
BL: Baseline
CarerQoL: Care-related Quality of Life instrument
CCMO: Central Committee on Research Involving Human Subjects; in Dutch: Centrale Commissie Mensgebonden Onderzoek
CONTR: Control
EQ-5D: EoroQoL-5D
EXP: Experimental
FMA: Fugl-Meyer Assessment
FU: Follow-up
GDPR: General Data Protection Regulation
ICF: International Classification of Functioning, disability and health
m: Month
MAL: Motor Activity Log
MAS: Modified Ashworth Scale
METC: Medical Research Ethics Committee (MREC); in Dutch: Medisch-Ethische ToetsingsCommissie (METC)
PI: Principal investigator
ReHab-TOAT: Remote Handling concept–based, Task-Oriented Arm Training
QoL: Quality of life
SD: Standard deviation
SPIRIT: Standard Protocol Items: Recommendations for Interventional Trials
SUS: System Usability Scale
TAM: Technology Acceptance Model
T-TOAT: Technology-assisted Task-Oriented Arm Training
UAT: Utrechtse Arm-hand Test
UTAUT: Unified Theory of Acceptance and Use of Technology
VAS: Visual analogue scale
WMO: Medical Research Involving Human Subjects Act; in Dutch: Wet Medisch-wetenschappelijk Onderzoek met mensen
