# Supplementary material for: Effects of a remote-handling-concept–based task-oriented arm training (ReHab-TOAT) on arm-hand skill performance in chronic stroke: a study protocol for a two-armed randomized controlled trial
Source: Trials. 2023 Mar 14;24:189. doi: 10.1186/s13063-023-07139-w (PMC10012705; doi:10.1186/s13063-023-07139-w)
Supplement: Supplementary file 1 — Additional file 1. SPIRIT checklist. [file 13063_2023_7139_MOESM1_ESM.docx]

# **SPIRIT Checklist for *Trials***

Complete this checklist by entering the page and line numbers where each of the items listed below can be found in your manuscript.

Your manuscript may not currently address all the items on the checklist. Please modify your text to include the missing information. If you are certain that an item does not apply, please state "n/a" and provide a short explanation. **Leaving an item blank or stating “n/a” without an explanation will lead to your manuscript being returned before review.**

Upload your completed checklist as an additional file when you submit to *Trials*. You must reference this additional file in the main text of your protocol submission. The completed SPIRIT figure must be included within the main body of the protocol text and can be downloaded here: [http://www.spirit-statement.org/schedule-of-enrolment-interventions-and-assessments/](about:blank)

In your methods section, please state that you used the SPIRIT reporting guidelines, and cite them as:

Chan A-W, Tetzlaff JM, Gøtzsche PC, Altman DG, Mann H, Berlin J, Dickersin K, Hróbjartsson A, Schulz KF, Parulekar WR, Krleža-Jerić K, Laupacis A, Moher D. SPIRIT 2013 Explanation and Elaboration: Guidance for protocols of clinical trials. BMJ. 2013;346:e7586

|  |  | **Reporting Item** | **Page and Line Number** | **Reason if not applicable** |
| --- | --- | --- | --- | --- |
| **Administrative information** | | | | |
| Title | [#1](about:blank#1) | Descriptive title identifying the study design, population, interventions, and, if applicable, trial acronym | Page 1 line 1-4, Page 4 line 72 |  |
| Trial registration | [#2a](about:blank#2a) | Trial identifier and registry name. If not yet registered, name of intended registry | Page 3 line 54, Page 4 line 72 |  |
| Trial registration: data set | [#2b](about:blank#2b) | All items from the World Health Organization Trial Registration Data Set | Page 1 line 54, Page 4 line 72 |  |
| Protocol version | [#3](about:blank#3) | Date and version identifier | Page 4 line 72 |  |
| Funding | [#4](about:blank#4) | Sources and types of financial, material, and other support | Page 4 line 72, Page 27 line 587-591 |  |
| Roles and responsibilities: contributorship | [#5a](about:blank#5a) | Names, affiliations, and roles of protocol contributors | Page 1 line 7-8, Page 4 line 72, Page 27 line 578-585 |  |
| Roles and responsibilities: sponsor contact information | [#5b](about:blank#5b) | Name and contact information for the trial sponsor | Page 4 line 72 |  |
| Roles and responsibilities: sponsor and funder | [#5c](about:blank#5c) | Role of study sponsor and funders, if any, in study design; collection, management, analysis, and interpretation of data; writing of the report; and the decision to submit the report for publication, including whether they will have ultimate authority over any of these activities | Page 4 line 72 |  |
| Roles and responsibilities: committees | [#5d](about:blank#5d) | Composition, roles, and responsibilities of the coordinating centre, steering committee, endpoint adjudication committee, data management team, and other individuals or groups overseeing the trial, if applicable (see Item 21a for data monitoring committee) | Page 24 line 541-542 | This is a one center study and only three employees of Adelante are working on this study. The main researcher JE, coordinating the whole study, is also the trained physical therapist providing the intervention. Therefore, there are no standard meetings. Meetings with the research coordinator are only scheduled, if necessary, e.g. if any problems occur during the research execution. |
| **Introduction** |  |  |  |  |
| Background and rationale | [#6a](about:blank#6a) | Description of research question and justification for undertaking the trial, including summary of relevant studies (published and unpublished) examining benefits and harms for each intervention | Page 5 line 74- page 8 line 157 |  |
| Background and rationale: choice of comparators | [#6b](about:blank#6b) | Explanation for choice of comparators | Page 6 line 126-128, Page 15 line 327, Page 16 line 371 |  |
| Objectives | [#7](about:blank#7) | Specific objectives or hypotheses | Page 8 line 159– Page 9 184 |  |
| Trial design | [#8](about:blank#8) | Description of trial design including type of trial (eg, parallel group, crossover, factorial, single group), allocation ratio, and framework (eg, superiority, equivalence, non-inferiority, exploratory) | Page 10 line 224 – Page 11 line 255 |  |
| **Methods: Participants, interventions, and outcomes** | | | | |
| Study setting | [#9](about:blank#9) | Description of study settings (eg, community clinic, academic hospital) and list of countries where data will be collected. Reference to where list of study sites can be obtained | Page 9 line 189–194 |  |
| Eligibility criteria | [#10](about:blank#10) | Inclusion and exclusion criteria for participants. If applicable, eligibility criteria for study centres and individuals who will perform the interventions (eg, surgeons, psychotherapists) | Page 10 line 212 – 222 |  |
| Interventions: description | [#11a](about:blank#11a) | Interventions for each group with sufficient detail to allow replication, including how and when they will be administered | Page 12 line 261 – Page 14 line 304 |  |
| Interventions: modifications | [#11b](about:blank#11b) | Criteria for discontinuing or modifying allocated interventions for a given trial participant (eg, drug dose change in response to harms, participant request, or improving / worsening disease) | Page 14 line 302 – Page 15 line 324 |  |
| Interventions: adherance | [#11c](about:blank#11c) | Strategies to improve adherence to intervention protocols, and any procedures for monitoring adherence (eg, drug tablet return; laboratory tests) | Page 9 line 189-194, Page 14 line 314 – Page 15 line 324 |  |
| Interventions: concomitant care | [#11d](about:blank#11d) | Relevant concomitant care and interventions that are permitted or prohibited during the trial | Page 10 line 230-231, Page 16 line 368 - 370 |  |
| Outcomes | [#12](about:blank#12) | Primary, secondary, and other outcomes, including the specific measurement variable (eg, systolic blood pressure), analysis metric (eg, change from baseline, final value, time to event), method of aggregation (eg, median, proportion), and time point for each outcome. Explanation of the clinical relevance of chosen efficacy and harm outcomes is strongly recommended | Page 15 line 326 – Page 17 line 376 |  |
| Participant timeline | [#13](about:blank#13) | Time schedule of enrolment, interventions (including any run-ins and washouts), assessments, and visits for participants. A schematic diagram is highly recommended (see Figure) | Page 11 line 237- 239, Page 16 line 371- Page 17 line 376 |  |
| Sample size | [#14](about:blank#14) | Estimated number of participants needed to achieve study objectives and how it was determined, including clinical and statistical assumptions supporting any sample size calculations | Page 18 line 400- Page 19 line 414 |  |
| Recruitment | [#15](about:blank#15) | Strategies for achieving adequate participant enrolment to reach target sample size | Page 9 line 196- 204 |  |
| **Methods: Assignment of interventions (for controlled trials)** | | | | |
| Allocation: sequence generation | [#16a](about:blank#16a) | Method of generating the allocation sequence (eg, computer-generated random numbers), and list of any factors for stratification. To reduce predictability of a random sequence, details of any planned restriction (eg, blocking) should be provided in a separate document that is unavailable to those who enrol participants or assign interventions | Page 11 line 241 - 255 |  |
| Allocation concealment mechanism | [#16b](about:blank#16b) | Mechanism of implementing the allocation sequence (eg, central telephone; sequentially numbered, opaque, sealed envelopes), describing any steps to conceal the sequence until interventions are assigned | Page 11 line 245-252 |  |
| Allocation: implementation | [#16c](about:blank#16c) | Who will generate the allocation sequence, who will enrol participants, and who will assign participants to interventions | Page 11 line 247-252 |  |
| Blinding (masking) | [#17a](about:blank#17a) | Who will be blinded after assignment to interventions (eg, trial participants, care providers, outcome assessors, data analysts), and how | Page 11 line 258-259 |  |
| Blinding (masking): emergency unblinding | [#17b](about:blank#17b) | If blinded, circumstances under which unblinding is permissible, and procedure for revealing a participant’s allocated intervention during the trial | n.A. | Participant blinding for the intervention is not possible, so unblinding is not needed. |
| **Methods: Data collection, management, and analysis** | | | | |
| Data collection plan | [#18a](about:blank#18a) | Plans for assessment and collection of outcome, baseline, and other trial data, including any related processes to promote data quality (eg, duplicate measurements, training of assessors) and a description of study instruments (eg, questionnaires, laboratory tests) along with their reliability and validity, if known. Reference to where data collection forms can be found, if not in the protocol | Page 15 line 327– Page 16 line 371, Page 18 line 378-383 |  |
| Data collection plan: retention | [#18b](about:blank#18b) | Plans to promote participant retention and complete follow-up, including list of any outcome data to be collected for participants who discontinue or deviate from intervention protocols | Page 15 line 321-322, Page 18 line 390-396 |  |
| Data management | [#19](about:blank#19) | Plans for data entry, coding, security, and storage, including any related processes to promote data quality (eg, double data entry; range checks for data values). Reference to where details of data management procedures can be found, if not in the protocol | Page 18 line 383–389 |  |
| Statistics: outcomes | [#20a](about:blank#20a) | Statistical methods for analysing primary and secondary outcomes. Reference to where other details of the statistical analysis plan can be found, if not in the protocol | Page 19 line 418 – Page 20 line 436 |  |
| Statistics: additional analyses | [#20b](about:blank#20b) | Methods for any additional analyses (eg, subgroup and adjusted analyses) | Page 19 line 420- 424 |  |
| Statistics: analysis population and missing data | [#20c](about:blank#20c) | Definition of analysis population relating to protocol non-adherence (eg, as randomised analysis), and any statistical methods to handle missing data (eg, multiple imputation) | Page 20 line 439-442 |  |
| **Methods: Monitoring** | | | | |
| Data monitoring: formal committee | [#21a](about:blank#21a) | Composition of data monitoring committee (DMC); summary of its role and reporting structure; statement of whether it is independent from the sponsor and competing interests; and reference to where further details about its charter can be found, if not in the protocol. Alternatively, an explanation of why a DMC is not needed | Page 20 line 451-456 |  |
| Data monitoring: interim analysis | [#21b](about:blank#21b) | Description of any interim analyses and stopping guidelines, including who will have access to these interim results and make the final decision to terminate the trial | n.A. | No interim analyses will be performed by the main researcher, because she is also the one who provides the intervention. End of the study is already known by start of this study, because a sample size calculation showed that 30 participants in each group are necessary. |
| Harms | [#22](about:blank#22) | Plans for collecting, assessing, reporting, and managing solicited and spontaneously reported adverse events and other unintended effects of trial interventions or trial conduct | Page 20 line 455- Page 21 line 465 |  |
| Auditing | [#23](about:blank#23) | Frequency and procedures for auditing trial conduct, if any, and whether the process will be independent from investigators and the sponsor | Page 20 line 451-454 |  |
| **Ethics and dissemination** | | | | |
| Research ethics approval | [#24](about:blank#24) | Plans for seeking research ethics committee / institutional review board (REC / IRB) approval | Page 20 line 445-449 |  |
| Protocol amendments | [#25](about:blank#25) | Plans for communicating important protocol modifications (eg, changes to eligibility criteria, outcomes, analyses) to relevant parties (eg, investigators, REC / IRBs, trial participants, trial registries, journals, regulators) | Page 20 line 447-450 |  |
| Consent or assent | [#26a](about:blank#26a) | Who will obtain informed consent or assent from potential trial participants or authorised surrogates, and how (see Item 32) | Page 9 line 197-205 |  |
| Consent or assent: ancillary studies | [#26b](about:blank#26b) | Additional consent provisions for collection and use of participant data and biological specimens in ancillary studies, if applicable | n.A. | Participant data will only be used for mentioned purposes of this study. No specimens will be collected. |
| Confidentiality | [#27](about:blank#27) | How personal information about potential and enrolled participants will be collected, shared, and maintained in order to protect confidentiality before, during, and after the trial | Page 18 line 378–389 |  |
| Declaration of interests | [#28](about:blank#28) | Financial and other competing interests for principal investigators for the overall trial and each study site | Page 28 line 699 |  |
| Data access | [#29](about:blank#29) | Statement of who will have access to the final trial dataset, and disclosure of contractual agreements that limit such access for investigators | Page 18 line 386–389 |  |
| Ancillary and post trial care | [#30](about:blank#30) | Provisions, if any, for ancillary and post-trial care, and for compensation to those who suffer harm from trial participation | n.A. | A standard research insurance for all participants is made prior to this study. |
| Dissemination policy: trial results | [#31a](about:blank#31a) | Plans for investigators and sponsor to communicate trial results to participants, healthcare professionals, the public, and other relevant groups (eg, via publication, reporting in results databases, or other data sharing arrangements), including any publication restrictions | Page 21 line 468-477 |  |
| Dissemination policy: authorship | [#31b](about:blank#31b) | Authorship eligibility guidelines and any intended use of professional writers | Page 27 line 579-585 |  |
| Dissemination policy: reproducible research | [#31c](about:blank#31c) | Plans, if any, for granting public access to the full protocol, participant-level dataset, and statistical code | Page 27 line 594-595 |  |
| **Appendices** | | | | |
| Informed consent materials | [#32](about:blank#32) | Model consent form and other related documentation given to participants and authorised surrogates | Page 28 line 600-601 |  |
| Biological specimens | [#33](about:blank#33) | Plans for collection, laboratory evaluation, and storage of biological specimens for genetic or molecular analysis in the current trial and for future use in ancillary studies, if applicable | n.A. | No biological specimens are collected during this study. |

It is strongly recommended that this checklist be read in conjunction with the SPIRIT 2013 Explanation & Elaboration for important clarification on the items. Amendments to the protocol should be tracked and dated. The SPIRIT checklist is copyrighted by the SPIRIT Group under the Creative Commons “[Attribution-NonCommercial-NoDerivs 3.0 Unported](about:blank)” license. This checklist can be completed online using https://www.goodreports.org/, a tool made by the EQUATOR Network in collaboration with Penelope.ai
